# Legal issues and underexplored data protection in medical 3D printing: A scoping review

**DOI:** 10.3389/fbioe.2023.1102780

**Published:** 2023-02-27

**Authors:** Ante B. V. Pettersson, Rosa Maria Ballardini, Marc Mimler, Phoebe Li, Mika Salmi, Timo Minssen, Ian Gibson, Antti Mäkitie

**Affiliations:** ^1^ Department of Otorhinolaryngology—Head and Neck Surgery, University of Helsinki and Helsinki University Hospital, Helsinki, Finland; ^2^ Department of Vascular Surgery, University of Helsinki and Helsinki University Hospital, Helsinki, Finland; ^3^ Department of Surgery, South Karelia Central Hospital, Lappeenranta, Finland; ^4^ Faculty of Law, University of Lapland, Rovaniemi, Finland; ^5^ The City Law School City, University of London, London, United Kingdom; ^6^ Sussex Law School, University of Sussex, Brighton, United Kingdom; ^7^ Department of Mechanical Engineering, Aalto University, Espoo, Finland; ^8^ Center for Advanced Studies in Biomedical Innovation Law (CeBIL), Faculty of Law, University of Copenhagen, Copenhagen, Denmark; ^9^ Department of Design, Production and Management, University of Twente, Enschede, Netherlands; ^10^ Research Program in Systems Oncology, Faculty of Medicine, University of Helsinki, Helsinki, Finland

**Keywords:** additive manufacturing, 3D printing, legal issues, legislation, regulation, medical, medicine

## Abstract

**Introduction:** 3D printing has quickly found many applications in medicine. However, as with any new technology the regulatory landscape is struggling to stay abreast. Unclear legislation or lack of legislation has been suggested as being one hindrance for wide-scale adoption.

**Methods:** A scoping review was performed in PubMed, Web of Science, SCOPUS and Westlaw International to identify articles dealing with legal issues in medical 3D printing.

**Results:** Thirty-four articles fulfilling inclusion criteria were identified in medical/technical databases and fifteen in the legal database. The majority of articles dealt with the USA, while the EU was also prominently represented. Some common unresolved legal issues were identified, among them terminological confusion between custom-made and patient-matched devices, lack of specific legislation for patient-matched products, and the undefined legal role of CAD files both from a liability and from an intellectual property standpoint. Data protection was mentioned only in two papers and seems an underexplored topic.

**Conclusion:** In this scoping review, several relevant articles and several common unresolved legal issues were identified including a need for terminological uniformity in medical 3D printing. The results of this work are planned to inform our own deeper legal analysis of these issues in the future.

## 1 Introduction

3D printing (3DP) or Additive Manufacturing (AM), a technology where various methods are used to build objects by successively adding layers of material, offers several possible medical applications (Salmi, 2021). Using plastic or metals as the raw material, medical devices such as inert implants, drill guides, tools or splints can be produced, often tailored to the individual patient’s anatomy to produce personalized medical devices. Conversely, using pharmacological substances as raw materials to produce drug formulations with a specific dosage and controlled release is known as pharmacoprinting, while using biomaterial and cells to produce tissue or organs is known as bioprinting. Although many of these applications are already in widespread use, confusion about medical device regulations has been identified among the barriers to further adoption (Pettersson et al., 2019). For an individual clinician, this may stem from unfamiliarity with legislation, the novelty of the field, as well as blurring of the traditional divide between device manufacturers and care providers. However, from a legal standpoint, several issues remain unsolved where legislation currently fails to provide a clear answer even if one had the expertise to ask the right questions. For instance, the liability of the designer of a personalized medical device—should the device fail—is an issue where underlying legal concepts may yet not have caught up with the reality of customized devices and distributed production.

Several reviews considering the technical foundation and clinical use of medical 3DP in specific subspecialties of medicine have been published (Chae et al., 2015; Youssef et al., 2015; VanKoevering et al., 2017; Wilcox et al., 2017). However, to the best of our knowledge, no previous review papers exist that examine legal issues specifically in medical 3DP. The aim of this scoping review was to identify specific controversies and unresolved legal issues in several areas of law related to medical 3DP. Besides pre-market regulations, the fields of liability, intellectual property rights (IPR), and data protection were chosen for exploration because of their known relevance to general 3DP or personalized medicine. While legislation is local, attempts have been made at harmonization between different jurisdictions, and medical devices and 3DP products are traded globally. Although in the present review we focus on an EU perspective, comparisons with other jurisdictions have been included as relevant.

## 2 Methods

In order to map out the currently existing literature and to identify and analyze knowledge gaps (Munn et al., 2018), a scoping review methodology was chosen. This choice of format was partly made because the qualitative nature of the subject and predicted non-uniform nature of publications was expected to preclude further attempts at synthesizing answers; and partly because our purpose was to let the results of this review inform future legal analysis. The review was guided by the PRISMA extension for scoping reviews (PRISMA-ScR) (Tricco et al., 2018). The process itself is described in detail in Table 1.

**TABLE 1:** Process of systematic literature search.

Database	PubMed	Scopus	Web of Science	Westlaw International
Search query	(((regulation) OR (regulations) OR (law) OR (liability) OR (legal) OR (jurisdiction) OR (legislation) OR (data protection) OR (intellectual property)) AND ((medical) OR (clinical) OR (surgery) OR (dentistry) OR (medicine) OR (surgical) OR (dental))) AND ((3D printing) OR (additive manufacturing) OR (rapid prototyping) OR (3DP) OR (3D-printing) OR (3-d printing))			((medical) OR (clinical) OR (surgery) OR (dentistry) OR (medicine) OR (surgical) OR (dental)) AND ((“3D printing”) OR (“additive manufacturing”) OR (“rapid prototyping")OR (3DP) OR (“3D-printing”) OR (“3-d printing"))
Results	614	258 (abstract-title-keyword)	383	384
After removing duplicates	959			
After screening abstracts	88			
After screening full texts	34			15

### 2.1 Data collection

PubMed, Web of Science and Scopus were chosen as the core databases for the review. Since it was discovered that these databases indexed only very few of the legal journals known to the authors where articles relevant to the theme were expected to have been published, the search was extended to legal databases. Searches were initially performed in Westlaw International, LexisNexis, HeinOnline and Social Sciences Research Network (SSRN). Of these, only Westlaw International was found to be compatible with the systematic methodology.

The core databases were searched with the query detailed in Table 1, the results exported into EndNote 20 (Clarivate), and duplicates removed. Westlaw International was searched with a similar query, and all full texts found were exported. All searches were performed between September and October 2020.

### 2.2 Study selection

Abstracts (available for PubMed, Web of Science and Scopus) were screened for inclusion by one author, after which full texts for selected articles were acquired and screened by the same person. For the legal database, full texts were divided up and screened for inclusion by individual authors. Articles that concerned legislation and patient-matched 3DP products were included, while articles focusing solely on pharmacoprinting, bioprinting, 3DP in forensic medicine or medical education, and mass-produced AM-manufactured devices used in the medical field were excluded. No restriction was placed on the date of publication. Publication types considered for inclusion were journal articles, book chapters and white papers, while blog posts and conference presentations were excluded. Potential articles found by the search were written in English, French, German, Mandarin, Russian or Spanish, all of which were screened by people fluent in the language.

### 2.3 Data extraction

A sample of five articles was read by all authors. On that basis, a spreadsheet encompassing relevant categories for findings was collaboratively developed. After that, data for all articles were abstracted by the first author, except in the case of articles not written in English, which were dealt with by authors fluent in the language as necessary.

## 3 Results

The review process, the number of initially identified articles, and the number of articles finally included in the review are detailed in Table 1, while a breakdown of excluded articles is presented in Table 2. Results are presented in Supplementary Table S1.

**TABLE 2:** Reasons for exclusion.

Exclusion criteria	Pubmed/Scopus/Web of Science	Westlaw International
not 3DP	453	43
non-medical 3DP	60	7
no legal aspects	334	
no access	10	
only education or forensic	12	
blog or transliterated speech		2
only bioprinting		18
only pharmacoprinting	16	4

The majority of the papers identified were in one way or another associated with pre-market approval, with post-market liability and IP rights occupying a smaller share, predominantly in the legal papers. Data protection was touched upon only in two papers (Feldman et al., 2018; Kritikos, 2018).

The legally undefined role of Computer Assisted Design (CAD) files was brought up by several papers, both in an IP as well as in a liability context (Colleen et al., 2015; Lindenfeld, 2016; Beck and Jacobson, 2017; Oudersluys, 2017; Dagne, 2020; Fairgrieve et al., 2020). From a liability point of view, controversy centers around whether the designer of a file can be held accountable for a defect in a medical device, since CAD files themselves may not be considered “products” in the legal sense of the word and are therefore not governed by product liability rules. For IP rights the key legal issue is whether CAD files in themselves are afforded any form of IP protection.

Confusion about the concept of the “manufacturer” of the “product”—even without the issue of CAD files—was mentioned in several papers (Colleen et al., 2015; Knight, 2016; Nissan, 2016; Beck and Jacobson, 2017; Pajot et al., 2019; Dagne, 2020; Manero et al., 2020).

Most publications concerning a specific subcategory of medical 3DP focused on implants. The classification of medical models under different jurisdictions was discussed in several papers and at least in the USA saw a change during the study period, with explicit Federal Drug Administration (FDA) guidance being published.

Looking at the timeline of publications, a key theme was the emerging distinction between custom-made and patient-matched personalized devices.

The different jurisdictions which the papers were concerned with included the USA, the EU, the United Kingdom, Canada, Australia, and to a lesser degree Russia, with the majority concerning the United States. While certain key regional differences were identified, the common unresolved legal issues as noted above were shared across jurisdictions.

## 4 Discussion

In this scoping review regarding legal issues surrounding medical 3DP, we identified the unclear legal status of Computer Assisted Design (CAD) files, the issue of who is the “manufacturer” of a 3DP product, the classification of medical models, and the definition of custom-made vs. patient-matched personalized devices as unresolved legal issues common to several jurisdictions. An additional finding was the surprising lack of papers concerning data protection.

The aim of the study was to identify these issues and we intend to address them more thoroughly in a separate paper in the future. Therefore, we will only present a short introduction to the issues and their relevant legal context below. We will also briefly address the methodological challenges faced while doing this review.

Data collection for the study ended in October 2020. The authors are aware of some papers within or adjacent to the scope of the study published since then, e.g., (Ballardini et al., 2022). However, since the end of the study period, the major development relating to legal issues in medical 3DP in the EU has been the eventual adoption of the MDR in May 2021. This process was postponed from the original date by the COVID-19 pandemic by a year, and has given rise to a number of issues, such as longer than expected waiting times for re-evaluating legacy devices from the MDD caused by too few “Notified Bodies” providing certification, unexpectedly strict requirements for evidence of “functional equivalency” with existing devices, and a delay in implementation of the EUDAMED database which holds an important role in the new regulatory scheme (Lucido, 2022). These issues will likely have an impact on the scope of medical devices available in the EU within the near future (Medtech Europe, 2022) and have prompted the EC to consider amendments to the legislation (Fimea, 2022). These unforeseen practical problems must also be considered when doing a more in-depth analysis of the issues outlined below.

### 4.1 Pre-market approval: Unstandardized terminology, lack of legislation for patient-matched devices, and unclear scope of exemptions

Medical device regulations are a fairly recent development. In the USA, the FDA was first granted the power to seize adulterated or misbranded medical devices in 1938. Later, with increasing complexity of products being brought on to the market, dealing retroactively with problematic devices was seen as inadequate. This resulted in the 1976 Medical Device Amendment changing the FDA’s role to proactively assessing and approving devices. In the EU, medical device regulations were for the first time harmonized in the 1990s, before that being considered national affairs (Masterson and Cormican, 2013).

While regulations in different jurisdictions are unlikely to fully converge, attempts are ongoing to harmonize medical device legislation globally. This movement, started in 1992 by the Global Harmonization Task Force, was later continued by the International Medical Device Regulators Forum (IMDRF) (International Medical Device Regulators Forum). Most countries have adopted some form of risk class based regulation, though the number and exact definition of risk classes (and indeed what is considered a medical device) may vary (Schuh and Funk, 2019). As an example, implantable or life-supporting products tend to be assigned a higher risk class (for example, Class II or Class III in the European classification) than band-aids (Class I in the European classification). In the EU, a major change happened during the study period with transition to the new Medical Device Regulation (MDR) from the previous Medical Device Directive.

Terminological confusion causes real-world issues when interfacing with regulators (Horst et al., 2019). In our review, terminology was found to be poorly defined. Besides the expected variation between “3D printing” and “additive manufacturing” for the technology, the terms “custom”, “custom-made”, “customized”, “patient-specific” and “patient-matched” were also used variably to mean different things.

The IMDRF suggests that “personalized medical device” be used as an umbrella term for custom-made, patient-matched and adaptable medical devices (International Medical Device Regulators Forum, 2018). “Custom-made” refers to devices specifically made in accordance with a written request by an authorized professional, under their responsibility, for a single patient. ‘Patient-matched’ refers to devices produced under the responsibility of the manufacturer, matched to patients based on patient anatomy, but produced by a uniform process that can be validated. Of note, the draft guideline used “patient-specific” as a synonym for “patient-matched”, but this was removed in the final document. One of the main issues discussed in the papers was the interface between “custom-made” and “patient-matched” devices.

The FDA echoes IMDRF terminology by defining “custom” devices, identical to the “custom-made” definition, as devices which fall under the custom device exemption, and “patient-matched devices” similarly to the IMDRF as medical devices matched to patient anatomy but that do not fall under custom device exemptions (FDA, 2017).

In the EU, however, while the term “custom-made” is explicitly present in the MDR, any mention of “patient-matched” devices or anything comparable is conspicuously absent. This has led to speculation as to whether all personalized devices may be considered custom-made in the EU (Kritikos, 2018). According to the MDR, “custom-made” refers to devices explicitly produced on the written instruction of a physician for an individual patient case but which do not need to fulfill the full EU pre-market approval requirements Official Journal of the European Union (2017). Moreover, devices “mass produced by means of industrial manufacturing processes” are not to be considered custom-made devices. This wording is quite vague, and was addressed in 2021 in a Q&A document by the EU’s Medical Device Coordination Group, which states that “[i]t must be underlined that products which are adaptable medical devices or patient-matched medical devices (as defined by IMDRF) are not qualified as CMDs and must follow the “standard” MDR regulatory pathway for placing on the market” and “A 3D printed device does not qualify as a CMD by default” (EU Medical Device Coordination Group, 2021). In some ways this merely brings us back to square one, with no specific concern given to regulation of patient-matched devices vs. traditional ones.

Some publications used “3D bioprinting”, sometimes inconsistently, to refer to regular medical 3DP (Kritikos, 2018; Schuh and Funk, 2019). However, a typical modern dictionary definition of bioprinting is “The use of 3D printing technology with materials that incorporate viable living cells” according to the Oxford Dictionary (Vermeulen et al., 2017).

Bioprinting as a term is not used in EU legislation. Instead, the Advanced Therapy Medicinal Products (ATMPs) legislation (separate from and predating the MDR) states that a Combined ATMP is one that incorporates an ATMP and a medical device. The same legislation also states that a Tissue Engineered Product (TEP) ATMP contains either living or dead tissue, but non-viable tissue is excluded from the definition if the principal mode of action of the pharmacologic, immunologic or metabolic (Official Journal Of The European Union, 2007).

Therefore, it seems that in the absence of viable cells, even complex biological products such as proteins and extracellular matrix can be used to produce a regular medical device as long as bioactive properties do not form the principal mode of action. However, when using non-viable tissues of human or animal origin, their harvesting and processing is regulated separately. Moreover, under the MDR (Annex VIII, 7.5, Rule 18), devices employing materials from these sources will be classified as class III unless they only come into contact with intact skin (Official Journal of the European Union, 2017). One may infer that biological material from plant, fungal or bacterial sources need not observe these restrictions.

A large number of legal articles in this study were excluded for exclusively focusing on bioprinting. While it seems wise to formulate regulatory approaches ahead of wide-scale adoption, bioprinting has currently seen no clinical use. Moreover, the legal issues are often very different from—and much more complex than—those of general medical 3DP.

A number of technical articles also touched upon the role of international standards in pre-market regulation. The foremost organizations publishing them are the International Organization for Standardization (ISO) and ASTM International (ASTM). For medical devices, relevant standards referenced by regulations include ISO13485 (Medical devices — Quality management systems — Requirements for regulatory purposes) and ISO10993 (Biological evaluation of medical devices), but several standards also exist specifically on 3DP, published by ISO Technical Committee 261 on Additive Manufacturing and ASTM Committee F42 on Additive Manufacturing Technologies. To date, one standard specifically addresses medical 3DP, namely, ISO—ASTM TR 52916:2022 (Additive manufacturing for medical - Data - Optimized medical image data).

### 4.2 Product liability: Who is a manufacturer and what are CAD files?

In addition to pre-market regulation requirements, manufacturers must also observe product liability laws. In the EU, these are not harmonized but generally follow the Product Liability Directive (PLD). The rationale for product liability laws is to disperse the risk of faulty products, so that instead of one unfortunate consumer having to suffer the consequences of a malfunction, the manufacturer will have to compensate the consumer, a risk that in turn will be reflected in the price of the product, thus spreading it among all consumers. The interplay between pre-market regulations and post-market liability is complex. In the United States, compliance with pre-market regulations may act as a full defense from potential liability claims against the manufacturer, whereas in the EU compliance provides only a partial defense. To establish liability under the Product Liability Directive, the injured person must prove the defect, the damage, and the causal link between the two (Kritikos, 2018). This is a form of “strict liability”, where it is enough to prove that a defect exists, regardless of whether it was caused by negligence. A product is considered defective for these purposes when it fails to provide the level of safety that a person is entitled to expect (Kritikos, 2018).

Important elements for current liability law include the “manufacturer” of a “product”. According to the PLD, a producer is defined as “the manufacturer of a finished product, the producer of any raw material or the manufacturer of a component part and any person who, by putting his name, trademark or other distinguishing feature on the product presents himself as its producer” (Council of the European Union, 1985). This means that all actors in the production chain may be considered liable, not just the one defined as ‘the manufacturer’ for pre-market approval purposes. However, with decentralized and complex production chains enabled by 3DP, it may be hard to identify potentially liable actors. A range of defects in 3DP products that would possibly lead to product liability claims includes defective imaging, defective original digital designs, defective digital files, defective printers, and defective materials. Whether the electronic blueprints of the product, known in the legal literature as Computer Assisted Design (CAD) files, are products or component parts that must comply with the PLD is not yet determined in a EU context (Fairgrieve et al., 2020), and similar issues also exist in the USA (Beck and Jacobson, 2017).

Human error would contribute to a defect. However, this falls under the negligence (tort) regime, which is separate from the PLD. Product liability is also distinct from medical negligence claims, and clinicians are expected to examine and review the quality of products before implantation. What kind of liability would apply to custom-made devices (Kritikos, 2018), or printing within hospitals, is unclear.

Strict liability as a legal principle originates in the USA but has since spread to several jurisdictions. A number of US papers included in this review proposed that, for the reasons outlined above, the concept itself does not work well for 3DP and that a return to the negligence standard for 3DP would make sense (Lindenfeld, 2016; Beck and Jacobson, 2017).

The current Product Liability Directive was adopted in 1985 and is therefore almost 40 years old. To address the changing nature of products on the market, including cases where software or AI comprises part of a product, in September 2022 the European Commission put forward a proposal for a new version of the PLD (European Commission, 2022). One of its recitals explicitly defines CAD files (in the sense of electronic files that control machine tools such as 3D printers) as products, subject to liability in cases where they are defective.

### 4.3 Intellectual property rights (IPR): CAD files again at the center of controversy

In general, 3DP technology has raised many legal issues about IP rights, medical 3DP being no exception. The legally undefined role of CAD files, besides the liability issues already noted, is also the crux of IPR legal issues. With 3D printers and raw materials widely available, the data file driving the printer, referred to as a CAD file in the legal literature, becomes the most valuable asset. However, whether CAD files are afforded legal protections is still undetermined. Moving from printed models of patient anatomy into tools, external aids and implants, legal issues over IP rights become increasingly relevant, and even more so if hospitals start manufacturing their own medical devices based on existing designs, as has been predicted (Laakmann, 2016; Jackson, 2017; Mukherjee et al., 2019).

It may be worth noting that what is called a CAD file in the legal literature when discussing 3DP is not necessarily the same as the technical definition. The legal definition tends to correspond to either what might technically be called a 3D object file (such as. stl) or a computer-aided manufacturing (CAM) file (such as. gcode). However, CAD files, as produced by modern CAD software, could be more akin to a full “technical package” and include elements such as tolerances, surface properties, materials, and so on—in short, all information necessary to manufacture and test the product. Comparing a 3D object file generated by an automatic process from scanned patient anatomy to this kind of technical package, one can see how they might be treated differently by legislation. Nevertheless, a CAD file is clearly an essential part of the 3DP process, so some form of IPR protection seems logical.

Several IPR protection mechanisms exist, such as copyright, patents, trademarks and design rights. Whether CAD files are currently afforded any of these protections remains unresolved. An alternative to IPR protection is trade secrets, where important data or details of a process are simply not disclosed (Esmond and Phero, 2015). However, unless truly revolutionary information is kept hidden, this can be quite simply overcome by reverse engineering, and is in general not compatible with an open science-based approach to medicine.

### 4.4 Data protection

Personalized medical products are based on the patient’s personal information, including information about diseases and pathologies and possibly anatomical data which may be identifiable even if explicit personal information has been removed. This necessitates compliance with data protection rules, such as the EU’s General Data Protection Regulation (GDPR). Data protection laws exist—*inter alia*—to safeguard personal data from being used without consent, a situation of increasing relevance with commercial applications of big data. Besides the risk of anonymous profiling, individual pieces of leaked medical data may also lead to targeted blackmail, as exemplified by the recent data breach involving an online psychotherapy provider (Ralston, 2021). Biometric data specifically diverted from the 3DP process could also be used for nefarious purposes, such as fooling facial recognition systems (Wang et al., 2018).

The scarcity of articles on data protection in the context of medical 3DP was an unexpected finding. In one of the articles included in the review, a shared drive open to everyone in the department, with no journaling of access, was chosen as the method for sharing CAD files (Pajot et al., 2019). Since imaging data for medical 3DP could be sensitive personal information both inside and outside of its medical implications, it needs to be treated with the same level of protection as other patient information if medical 3DP is to become routine. An interesting observation was that access to actual physical artifacts may need to be controlled as well (Feldman et al., 2018). Bringing 3DP production in-house helps resolve these data protection-related legal issues since dealing with patient information is a core activity for hospitals. Even so, existing electronic health record (EHR) and picture archiving and communication systems (PACS) may need further development to effectively support a secure 3DP workflow.

### 4.5 Methodological challenges

To our knowledge this paper is the first systematic literature search on the topic of legal issues surrounding or arising from medical 3DP. Systematic search methodologies (including systematic reviews, scoping reviews, topical reviews and critical reviews) (Powell and Koelemay, 2022), while the mainstay of medical research, are not common in the legal field. This became evident while extending our search into legal databases. Searches were initially performed in four legal databases: Westlaw International, LexisNexis, HeinOnline, and Social Sciences Research Network (SSRN). Only the results from Westlaw International were eventually used since the user interface of that database was the only one where a workflow compatible with a systematic process could be established, though it also required discussions with the database helpdesk and some non-intuitive workarounds. In general, the interfaces with legal databases were found not to support systematic review, with poor export of metadata to be used in reference management systems, an inability to export more than a few references at a time, among other issues. The interface with SSRN did not support Boolean searches at all, precluding any further usage. The legal databases also did not contain abstracts, but only the full text. The advantage of this was that any paper indexed was accessible, but it also meant that the majority of potential “hits” were just a word mentioned in passing, such as in a footnote. Though cumbersome, the end results of the legal database search were found to be non-overlapping and complementary to the articles found in the natural science/medical databases. A number of simple changes to search interfaces would greatly facilitate future interdisciplinary research in the legal and social science fields.

## 5 Conclusion

In this scoping review of legal challenges in medical 3DP, we identified several common unresolved legal issues. Most prominent among these were terminological confusion, especially regarding the difference between custom-made and patient-matched medical devices, as well as a lack of specific legislation for patient-matched devices, the issue of who is considered a manufacturer for liability purposes, and the legally undefined role of CAD files both from a liability as well as from an IPR perspective. A lack of publications on data protection in medical 3DP was also noted. These issues warrant further scholarly legal exploration, which we mean to do in a separate future paper. Lack of support for systematic search and review methodology in common legal databases was noted as a hindrance for interdisciplinary reviews such as the present one.
